# Endoscopic “combined-arms approach” for the management of a giant intraductal papillary mucinous neoplastic lesion of the bile duct

**DOI:** 10.1055/a-2545-2606

**Published:** 2025-03-12

**Authors:** Theodor Voiosu, Andrei Mihai Voiosu, Mihaela Maria Birligea, Andrea Tringali, Istvan Hritz, Bogdan Mateescu, Marc Barthet

**Affiliations:** 1 Department of Gastroenterology, Colentina Clinical Hospital, Bucharest, Romania; 2 Carol Davila University of Medicine and Pharmacy, Bucharest, Romania; 3 Digestive Endoscopy Unit, Fondazione Policlinico Gemelli IRCCS – Catholic University, Rome, Italy; 4 Centre for Endoscopic Research Therapeutics and Training (CERTT), Universita Cattolica del Sacro Cuore, Rome, Italy; 5 37637 Centre for Therapeutic Endoscopy, Semmelweis University, Budapest, Hungary; 6 Department of Gastroenterology, Hopital Nord, Marseille, France; 7 128791 Faculty of Medicine, Aix-Marseille University, Marseille, France

An intraductal papillary mucinous neoplastic lesion (IPMN) of the bile duct is a rare, difficult-to-treat disease causing complex strictures of the biliary tree. For patients who are not amenable to radical surgery, maintaining patent bile ducts requires an individualized approach, with endoscopists sometimes resorting to outside-the-box solutions to provide long-term drainage 1 . We report the case of a 77-year-old patient failing conventional drainage with multiple plastic stents who presented with recurrent bouts of cholangitis and a newly developed intrahepatic cystic lesion ( Fig. 1 )

**Fig. 1:**
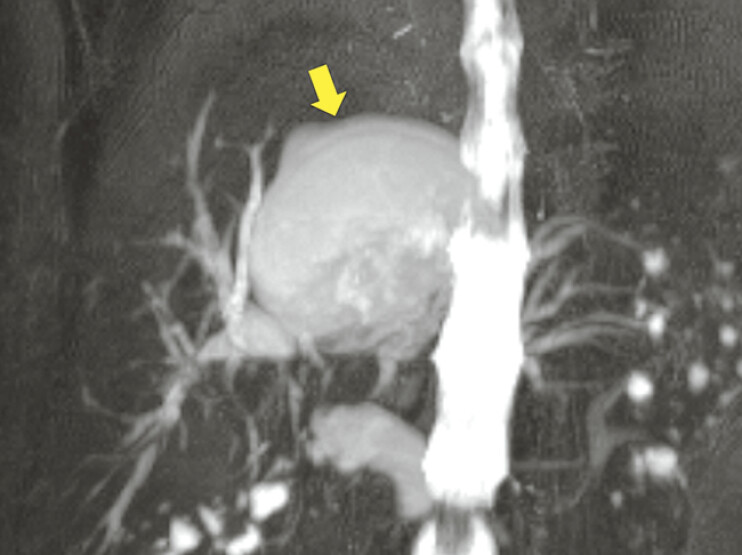
Magnetic resonance cholangiopancreatography showing a large intrahepatic cystic lesion (arrow) and dilated intrahepatic bile ducts.

An endoscopic ultrasound evaluation was performed that showed a large fluid collection in the left liver lobe with multiple intracystic nodules and dilated upstream bile ducts. We performed a cystogastrostomy with a 20-mm lumen-apposing metal stent (LAMS) to resolve the collection and improve biliary drainage ( Fig. 2 ). On follow-up computed tomography imaging, cyst resolution was confirmed ( Fig. 3 ) and the LAMS was extracted after one month, with subsequent bilateral intraductal radiofrequency ablation followed by placement of self-expandable metal stents into the left and right hepatic ducts ( Video 1 ) through endoscopic retrograde cholangiography. At the one-year follow-up the patient was asymptomatic, having required only one additional endotherapy session with stent-in-stent placement of two plastic stents for recurring cholangitis due to stent ingrowth.

**Fig. 2:**
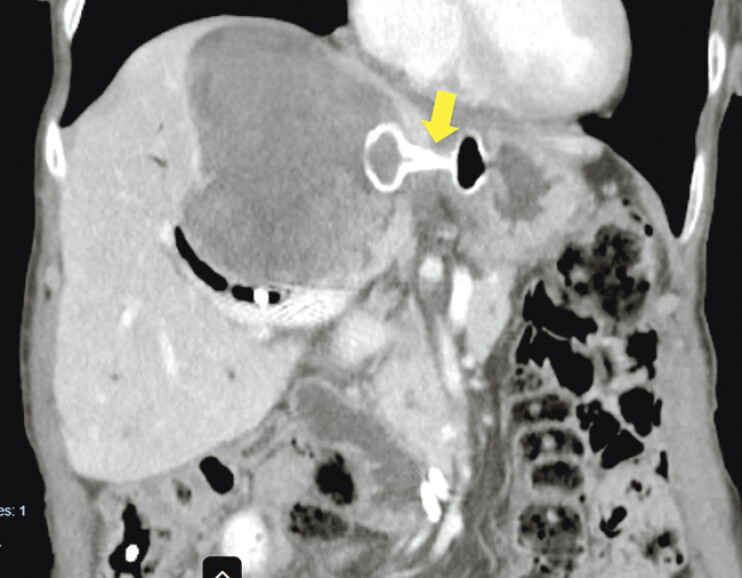
Computed tomography (CT) scan showing a correctly placed 20-mm lumen-apposing metal stent (arrow) connecting the intrahepatic cyst and the stomach.

**Fig. 3:**
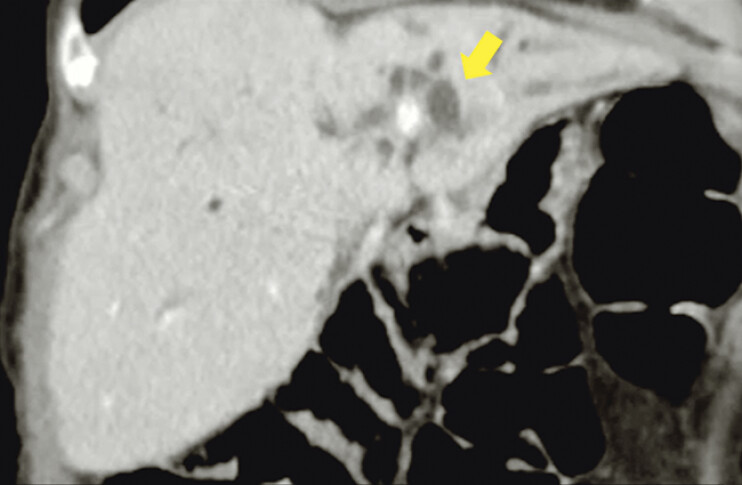
Follow-up CT scan showing resolution of the cystic lesion with persistent dilatation (arrow) of the bile ducts.

The steps of the endoscopic “combined-arms approach” – endoscopic retrograde cholangiography and endoscopic ultrasound-guided drainage of the giant intraductal papillary mucinous neoplastic lesion of the bile duct.Video 1

This case highlights the importance of tailoring endoscopic solutions to address the unique challenges caused by inoperable IPMNs of the bile duct that may frequently require a progressive step-up in a “combined-arms” tactic, including both intraductal and transmural approaches to optimize biliary drainage.

Endoscopy_UCTN_Code_TTT_1AS_2AH

## Notes

Endoscopy E-Videos https://eref.thieme.de/e-videos
*E-Videos* is an open access online section of the journal *Endoscopy* , reporting on interesting cases and new techniques in gastroenterological endoscopy. All papers include a high-quality video and are published with a Creative Commons CC-BY license. Endoscopy E-Videos qualify for HINARI discounts and waivers and eligibility is automatically checked during the submission process. We grant 100% waivers to articles whose corresponding authors are based in Group A countries and 50% waivers to those who are based in Group B countries as classified by Research4Life (see: https://www.research4life.org/access/eligibility/ ). This section has its own submission website at https://mc.manuscriptcentral.com/e-videos .
